# Primary health care coverage in Brazil: a dataset from 1998 to 2020

**DOI:** 10.1186/s13104-023-06323-0

**Published:** 2023-04-25

**Authors:** Ronaldo Fernandes Santos Alves, Cristiano Siqueira Boccolini, Lais Ribeiro Baroni, Patricia de Moraes Mello Boccolini

**Affiliations:** 1 Institute of Scientific and Technological Communication and Information in Health. Oswaldo Cruz Foundation, Fiocruz, Rio de Janeiro, Brazil; 2 Federal Center for Technological Education of Rio de Janeiro, CEFET/RJ, Rio de Janeiro, Brazil; 3 Center for Information, Public Policies and Social Inclusion, Petropolis Medical School, FMP/UNIFASE, Petrópolis, Brazil

**Keywords:** Brazil, Primary Health Care, Family Health Strategy, Population Characteristics, Socioeconomic Factors, Routinely Collected Health Data, Health Information Systems, Database, Metadata

## Abstract

**Objectives:**

Primary health care builds the backbone of an effective healthcare system and can improve population health, reduce cost growth, and lessen inequality. We offer a machine-readable and open-access dataset on primary health care coverage in Brazil from 1998 to 2020. This dataset is interoperable with epidemiological data from two major studies and reusable by the research community worldwide for other purposes, such as monitoring progress toward universal health coverage and studying the association between primary health care and health outcomes.

**Data description:**

The dataset gathers official and public information from the “e-Gestor AB” platform of the Ministry of Health of Brazil and restricted data obtained by the Brazilian Access to Information Law. It includes 1,509,870 observations and 35 attributes aggregated by months/years and policy-relevant geographic units (country, macroregions, states, municipalities, and capitals) on primary health care team count and their absolute and relative population coverage estimates, information on the More Doctors Program implementation and physician counts, and spatial, demographic, and socioeconomic characteristics. We automated all data processing and curation in the free and open software R. The codes can be audited, replicated, and reused to produce alternative analyses.

## Objective

Primary Health Care (PHC) builds the backbone of an effective healthcare system and can improve population health, reduce cost growth, and lessen inequality [1]. PHC provides an overall framework for organizing and delivering care that best meets the needs and circumstances of individuals, families, and communities. It should address physical, mental, and social health and wellbeing based on person-centeredness, continuity, comprehensiveness, and care integration principles [2, 3]. PHC is one of the main pathways to achieving universal health coverage, which is the Sustainable Development Goals target 3.8 and a Brazilian national priority [4].

Brazil is the only country with more than 100 million inhabitants that guarantees the human right to health through universal and free access to health services. The Family Health Teams (FHT) are the principal strategy for delivering community-based primary care in Brazil’s Unified Health System – known as “Sistema Único de Saúde” or SUS. Until June 2007, only FHT formed PHC teams. After that, parametrized Basic Health teams to adhere to the National Program for Access and Quality Improvement in Primary Care (pBHT), and Basic Health teams equivalent to the FHT (eBHT) became part of the PHC teams.

We offer a machine-readable and open-access dataset on PHC coverage in Brazil from 1998 to 2020. It also contains information about the More Doctors Program (MDP), which seeks to supply health care providers in underserved areas [5]. This dataset is interoperable with epidemiological data from two major studies [6–9] – to correlate PHC with child mortality, hospitalization, immunization, and breastfeeding – and reusable by the research community worldwide for other purposes, such as monitoring progress toward universal health coverage.

## Data description

The dataset gathers official and public information from the “e-Gestor AB” platform of the Ministry of Health of Brazil (MoH) [10] and restricted data obtained by the Brazilian Access to Information Law (LAI). We manually extracted the public data on December 06, 2021. It includes the monthly count of PHC teams and their absolute and relative population coverage estimates. The restricted data provided monthly information on the MDP implementation and physician counts. We add previously consolidated spatial, demographic, and socioeconomic data, which are described and available elsewhere [9]. The final dataset has 1,509,870 observations and 35 attributes aggregated by months from 1998 to 2020 and policy-relevant geographic units (country, macroregions, states, municipalities, and capitals). We automated all data processing/curation in the free and open software R. The codes can be audited, replicated, and reused to produce alternative analyses.

Table 1 provides an overview of the files and datasets available in Synapse [11]. Data files 1–2 hold the codes for ingestion, transformation, and loading routines. Dataset 1 includes PHC’s raw files, and datasets 2–3 comprise workflow endpoints with the PHC and MDP data. Data file 3 builds the final dataset, which was integrated, harmonized, and enriched with spatial, demographic, and socioeconomic data [9]. The HTML files show type-specific information for intermediate and final datasets attributes, including statistical summaries and missing frequencies (data files 4–6). Data file 7 documents the metadata and attribute descriptions of the final dataset (dataset 4).

**Table 1:** Overview of data files/data sets

Label	Name of data file/data set	File types (file extension)	Data repository and identifier
Data file 1	script_phc_ingestion	R code (.r)	Synapse: 10.7303/syn26529247 [11]
Data file 2	script_pmm_ingestion	R code (.r)	Synapse: 10.7303/syn26529247 [11]
Data file 3	script_master_phc	R code (.r)	Synapse: 10.7303/syn26529247 [11]
Data file 4	phc_sprint_dataselfie	HTML (.html)	Synapse: 10.7303/syn26529247 [11]
Data file 5	pmm_sprint_dataselfie	HTML (.html)	Synapse: 10.7303/syn26529247 [11]
Data file 6	phc_master_dataselfie	HTML (.html)	Synapse: 10.7303/syn26529247 [11]
Data file 7	phc_master_overview	excel (.xlsx)	Synapse: 10.7303/syn26529247 [11]
Dataset 1	phc_data_raw	zipped (.zip)	Synapse: 10.7303/syn26529247 [11]
Dataset 2	phc_clean_data	R data (.rdata)	Synapse: 10.7303/syn26529247 [11]
Dataset 3	pmm_clean_data	R data (.rdata)	Synapse: 10.7303/syn26529247 [11]
Dataset 4	phc_master_data	R data (.rdata)	Synapse: 10.7303/syn26529247 [11]

Anyone can browse the content on the Synapse website, but you must register for an account using your email address to download the files and datasets.

### Data construction

The data workflow involves two main steps. The first step covered ingestion, transformation, and loading routines of PHC and MDP data. We downloaded three raw files from the “e-Gestor AB” platform with the number of PHC teams by municipalities and months/years and received one raw file via LAI with the MDP physician’s names and activity locations and periods. The essential features of data transformation were (i) variables selection/renaming and observations filtering, (ii) correction of codes and names identifying geographic units, (iii) cleansing numeric values, e.g., excluding special characters, (iv) cleansing inconsistent dates, e.g., exchanging start/end activities dates, (v) converting MDP individual data to ecological data and flagging municipalities with the implemented program, and (vi) enrichment of the municipal datasets with data aggregated by states, macroregions, and country. This step produced two datasets treated and usable in the final dataset construction.

The second step comprises data integration, harmonization, and enrichment with spatial, demographic, and socioeconomic characteristics [9]. We combined the treated datasets according to the months/years and codes of geographic units. The PHC’s absolute coverage estimates followed the MoH method: number of FHT × 3,450 + (number of pBHT + number of eBHT) × 3,000. Besides, the PHC’s relative coverage estimates considered the total population size in the current year. The absolute estimates will equal the population size whenever the numerator value equals or exceeds the denominator value; in turn, the maximum values of the relative estimates were 100%. Remarkably, the PHC’s indicators do not include the physicians from the MDP. R codes and data processing/curation were peer-reviewed, and their results compared to the official site’s information.

## Limitations

We should mention the potential limitations and usages of the dataset. First, the PHC’s indicators measure the human resources available in PHC for a geographically defined population. Therefore, a higher PHC coverage indicates a higher potential for offering essential health services to the people and better accessibility to this level of care. However, these indicators do not effectively measure the amount or quality of work performed by PHC teams or their services. Information on the quantity and quality of care provided complements the results of the PHC coverage and is available in other Brazilian Health Information Systems. Second, conclusions about time-trend analysis need to be interpreted with caution since there was a methodological change in calculating the PHC’s indicators in July 2007, including the FHT, eBHT, and pBHT. Additionally, information on the MDP is available for the program duration period (2013–2018). Third, the parameters in calculating PHC indicators reflect an average number of individuals covered by these teams according to the Brazilian National Primary Health Care Policy, not the number of people effectively registered/assigned to PHC teams. Fourth, our PHC’s relative coverage estimates considered the total population size in the current year and may diverge somewhat from MoH estimates, which consider the population size in the previous year. Finally, we could only build the dataset at an ecological level and did not incorporate the 2021 data because the definition of PHC teams has changed from this year in Brazil.

## Data Availability

The data described in this Data note can be freely and openly accessed on Synapse under DOI: 10.7303/syn26529247. Anyone can browse the content on the Synapse website, but you must register for an account using your email address to download the files and datasets. Please see Table 1 and references [6, 7, 11] for details and links to the data.

## Abbreviations

eBHT: Basic Health Teams Equivalent to the Family Health Teams
FHT: Family Health Teams
LAI: Brazilian Access to Information Law
MDP: More Doctors Program
MoH: Ministry of Health
pBHT: Parametrized Basic Health Teams to adhere to the National Program for Access and Quality Improvement in Primary Care
PHC: Primary Health Care
SUS: Brazil’s Unified Health System
